# Unraveling the Mysteries of Autoimmune Gastritis

**DOI:** 10.5152/tjg.2024.24563

**Published:** 2024-11-25

**Authors:** İrfan Soykan, Ramazan Erdem Er, Yigit Baykara, Cağdaş Kalkan

**Affiliations:** 1 Ankara University Medical School, İbn-i Sina Hospital, Gastroenterology, Ankara, Türkiye; 2 Department of Pathology, Stanford Medicine, Transfusion Medicine and Blood Banking, California, USA; 3 Department of Gastroenterology, Ministry of Health, Bilkent City Hospital, Çankaya, Ankara, Türkiye

**Keywords:** Anti-parietal cell antibody, autoimmune gastritis, gastrin, neuroendocrine tumor type 1

## Abstract

Autoimmune gastritis is an immune-mediated disease characterized by the destruction of parietal cells and atrophy of the oxyntic mucosa due to anti-parietal cell antibodies. It may lead to serious conditions including iron/vitamin B_12_ and micronutrient deficiencies, neurological disorders, and gastric malignancies. The exact mechanism of this disease is not exactly understood; however, dysregulated immunological mechanisms appear to be major contributors. Patients with this disease are often asymptomatic but may present with gastrointestinal symptoms and/or iron/vitamin B_12_ deficiencies. Although important serological markers are available and despite advanced endoscopic techniques, the definitive diagnosis relies on histopathological examination of gastric corporal biopsy specimens. Autoimmune gastritis is closely related with increased risk of gastric neuroendocrine tumors and gastric adenocarcinoma. Patients with autoimmune gastritis do not benefit from specific treatments, thus, management is directed to restore micronutrient deficiencies and to prevent occurrence of neoplastic transformation with appropriate endoscopic surveillance.

**Main Points**

- *Helicobacter pylori* infection contributes to the pathogenesis of autoimmune gastritis (AIG).
- Many patients with AIG are asymptomatic, though they often present with dyspepsia and/or signs of micronutrient deficiencies.
- The diagnosis of AIG relies on a combination of serological markers and histopathological findings.
- Useful markers for the diagnosis of AIG in patients with atrophy include antiparietal cell antibodies, serum gastrin levels, pepsinogen I/II ratio, vitamin B_12_ levels, and anti-*H. pylori* IgG.
- The incidence of type 1 neuroendocrine tumors developing in AIG patients is 4.7% over a 2-year follow-up period.
- Risk factors for gastric cancer development include age over 60 years, intestinal metaplasia without pseudopyloric metaplasia, and vitamin B_12_ deficiency.
- Although there is no definitive treatment for AIG, management focuses on correcting iron and vitamin deficiencies, as well as ensuring the early detection of pre-neoplastic lesions through regular endoscopic monitoring.

## Introduction

Autoimmune gastritis (AIG) is a chronic, autoimmune disorder that is caused by inflammation in the stomach, leading to the gradual destruction of parietal cells.^1^ In cases of AIG, anti-parietal cell antibodies (APCA) specifically target the H^+^/K^+^ ATPase, resulting in the destruction of parietal cells by autoreactive T cells. Over time, this process leads to the gradual atrophy of the oxyntic glands within the mucosa. Anti-intrinsic factor antibodies may also occur and lead to a decrease in intrinsic factor levels. The destruction of the parietal cells causes decreased gastric acid secretion which in turn causes chronic hypochlorhydria and ultimately, achlorhydria. Decreased or absent levels of hydrochloric acid (HCl) and intrinsic factor have a marked effect on gastrointestinal (GI) function, such as the deficiency of iron, vitamin B_12_, and other micronutrients.^2^ In addition, it can cause upper GI symptoms such as nausea, vomiting, epigastric pain, and bloating.^3^ The chronic hypo/achlorhydric state stimulates the release of gastrin, which causes hypergastrinemia and enterochromaffin-like (ECL) cell hyperplasia that increases the risk of type 1 carcinoid tumors of the stomach.^4^ The goal of this review is to offer an extensive overview of AIG, describing the latest data on pathogenesis, clinical presentation, diagnosis, and management options of this entity as well as to discuss potential future directions in light of existing literature. This review also focuses on the complications and follow-up strategies in this group of patients, current pharmacological agents, and dietary and lifestyle modifications available in this clinical setting.

## Epidemiology

Autoimmune gastritis is a significant health concern with increasing prevalence; however, the epidemiological data on AIG remain limited and its true prevalence may be underestimated. Estimates of AIG prevalence range from 0.5% to 4.5%, influenced by variables like the population studied, age, gender, and ethnic background.^5^

## Pathogenesis

Although the precise cause of AIG is not well established, genetic and environmental factors are responsible for its occurrence. Although the involvement of APCA and anti-intrinsic factor antibodies in AIG is not fully established, H+/K+-ATPase reactive CD4 T-cells are recognized as the main culprits driving the autoimmune reaction. It is believed that T-cell activity leads to prolonged stimulation of B lymphocytes, which in return produces APCA and anti-intrinsic factor antibodies.^6^ Some studies suggest that the destruction of the parietal cells relies on Th1 CD4 T-cells and Fas/Fas-ligand interactions, either via interaction between CD4 T-cells and parietal cells with increased Fas expression or through cell-to-cell interactions between parietal cells.^7^ Lahner et al^8^ reported that there should be a genetic predisposition, and some reports have proposed a relationship with HLADRB103 and HLA-DRB104. The molecular mimicry among *Helicobacter pylori* antigens and H+/K+-ATPase has been raised as a responsible pathway because of the high homology between the beta subunit of *H. pylori* urease and the beta subunit of ATPase.^9^ The proton pump located on parietal cells is the main target autoantigen known by APCAs. APCA targets both the alpha subunits and beta subunits of the proton pump.^10^ Autoreactive T helper 1 cells and cytotoxic T cells, which are seen in the mucosa, play a major pathogenetic role in AIG.^6^ These activated autoreactive T cells produce pro-inflammatory cytokines such as TNF-alpha and INF-gamma that amplify the immune response and cause parietal cell apoptosis. Extensive tissue remodeling, which is sustained by gastric myofibroblasts, leads to gastric atrophy of the oxyntic mucosa.^7^ Environmental factors such as *H. pylori* infection and exposure to certain dietary components can activate the autoimmune response in genetically susceptible individuals. Particularly, molecular mimicry may activate autoreactive immune cells targeting the gastric mucosa.^11^ *H. pylori* infection may contribute to the autoimmune process through molecular mimicry, particularly in those carrying specific toll-like receptors such as TLR5 rs574417C-allele.^12^ The molecular mimicry between *H. pylori* antigens and gastric H+/K+-ATPase has been accepted as a pathogenic mechanism, due to the high homology seen between the beta subunit of *H. pylori* urease and the beta subunit of ATPase.^9^ Hypo/achlorhydria bypasses the somatostatin-induced negative feedback that adjusts gastrin secretion by the antral G-cells. Ultimately, the achlorhydric medium increases gastrin-17 levels and causes gastric flora alterations. This process promotes the occurrence of gastric lesions such as type 1 gastric neuroendocrine tumors (NETs). During the course of the disease, atrophy in the body and fundus becomes widespread, achlorhydria occurs, and ECL cell hyperplasia that was linear at the beginning becomes micronodular and then turns into the type 1 gastric NETs in the last stage.^13^

## Clinical Manifestations and Diagnosis

In the early stages, autoimmune gastritis tends to be asymptomatic, with no distinct symptoms linked directly to the condition, often leading to delays in diagnosis. While the clinical range of symptoms of AIG has been described, there remain challenges in identifying its diverse clinical manifestations. Many patients, despite being asymptomatic, present with dyspepsia and heartburn. Symptoms may occur periodically and may be aggravated or alleviated by some foods. Soykan et al^14^ stratified 109 patients into 3 groups based on symptom patterns: group 1 included abdominal symptoms (47%); group 2 included symptoms caused by iron or vitamin B_12_ deficiencies (33%); and group 3 included non-specific symptoms (20%). They also concluded that AIG is more prevalent in women (66%) than men. The most common reasons for seeking medical attention in AIG patients are usually abdominal bloating and deficiencies in iron or vitamin B_12_, often following vague symptoms such as intermittent diarrhea. However, symptoms may vary over time. The same group investigated 165 AIG patients in order to define gastric emptying time and reported that 70% of patients had upper GI symptoms at that time.^15^ Carabotti et al^16^ investigated 779 patients with AIG by means of the GI symptom profile and evaluated if symptomatic patients are delineated by specific clinical features. They concluded that 60% of cases were related to dyspeptic symptoms, in which most of the patients were young females, did not have smoking habits and without vitamin B_12_ deficiency. In light of the current evidence, we can conclude that 60% of patients with AIG have dyspeptic symptoms. The pathogenesis of dyspeptic symptoms is not known exactly. It is essential to note that, in clinical practice, some patients with AIG present with heartburn and regurgitation suggesting acid reflux, although these patients have hypo/achlorhydria instead. In fact, these patients are often given gastric acid reducing agents. However, no information exists on the potential role of acid reflux in the initiation of the symptoms, and acid reducing agents may not be suitable for that reason. In order to shed light on this issue, Tenca et al^17^ investigated 41 patients with AIG by using 24-hour pH measurement and concluded that acid reflux rarely occurred, suggesting that heartburn and regurgitation may be attributable to non-acid reflux, or the patients may have functional heartburn. On the other hand, Pilotto et al^18^ investigated 38 AIG patients using high resolution manometry and 24-hour pH measurement. In their study, they demonstrated objective signs of acid reflux in 5% of patients, and in most of the patients, reflux symptoms had a functional origin. In this condition, we can presume that proton pump inhibitors would not be helpful in symptom resolution in which acid secretion is virtually decreased or absent. Nevertheless, hypochlorhydria and subsequent hypergastrinemia may cause delayed gastric emptying, causing abdominal distention and early satiety. In this context, Kalkan et al^15^ investigated 165 patients with AIG by means of a scintigraphic gastric emptying test and found that 80% of AIG patients had delayed gastric emptying of solid food. However, a subset of patients with upper GI symptoms had substantial delayed gastric emptying in contrast to subjects with systemic symptoms. In this study, parameters that affected gastric emptying time were serum gastrin levels, chronic inflammation, and an increase in the degree of atrophy of the mucosa. This is an important finding because, in the differential diagnosis of delayed gastric emptying, AIG may be an important entity. There might be some other motor disturbances in AIG patients due to hypergastrinemia, such as altered gallbladder function which may cause GI symptoms. Gallbladder dysmotility may cause vague upper abdominal pain that is not distinguished from other functional GI disorders. Although most subjects with gallbladder dysmotility have right upper quadrant pain, some of them present with poorly defined chronic abdominal pain and distention. Yakut et al^20^ assessed 41 patients with AIG and 29 healthy controls using abdominal ultrasonography with the ellipsoid method. They measured both fasting and postprandial gallbladder volumes and calculated the gallbladder ejection fraction from these volumes. The results showed that AIG patients had a lower gallbladder ejection fraction and there were no significant differences in mean fasting and postprandial gallbladder volumes between the patients and the control group. Regression analysis indicated that “abdominal bloating” was a risk factor for reduced gallbladder ejection fraction in AIG patients. The authors concluded that some upper gastrointestinal symptoms in these patients might be attributed to gallbladder dysmotility.^19^ Since marks of autonomic nervous system alterations occur in 24%-100% of patients with autoimmune diseases.^20^ and some of the patients with AIG may have delayed gastric emptying and altered gallbladder motor function causing upper GI symptoms, this condition may be associated with autonomic nerve dysfunction. Kalkan et al^21^ investigated 75 patients diagnosed with AIG by parasypmpathic and sympathic nervous system tests and synthintraphic gastric emptying test. They identified 62 patients with parasypmpathic and sympathic nervous system dysfunction and concluded that there is a strong link between dysfunction and delayed gastric emptying. In humans, ghrelin is produced in the body and fundus of the stomach, promoting gastric emptying through its effects on the vagus nerve. Motilin which is released by duodenal endocrine cells, enhances gastric motility by stimulating the cholinergic nerve endings located in the myenteric plexus. Given that ghrelin and motilin are key mediators of gastric emptying and that some AIG patients may experience delayed gastric emptying, causing upper GI symptoms, Kalkan and Soykan^22^ explored the roles of ghrelin and motilin in AIG patients with delayed gastric emptying and autonomic nerve dysfunction. They concluded that ghrelin and motilin concentrations were significantly reduced in AIG patients with delayed gastric emptying and altered autonomic function. The reduction of ghrelin and plasma motilin concentrations in AIG suggests their possible involvement in the delayed gastric emptying seen in those patients.

Since AIG is an autoimmune disease, it is possible that AIG may be related to other autoimmune diseases. Kalkan and Soykan investigated the factors connected with other autoimmune disorders to figure out which factor(s) estimate the existence of another autoimmune disease in patients with AIG.^23^ They concluded that 50% of patients with AIG had another autoimmune illness associated with polyautoimmunity. Polyautoimmunity found in patients with AIG was associated with the presence of ECL cell hyperplasia, serum gastrin, and serum chromogranin A levels. Physicians dealing with this kind of disorder should assess AIG patients for the presence of polyautoimmunity.

It is clinically important to mention that some patients with AIG do not exhibit APCA in their serum. In such a case, APCA is not a requisite for verifying AIG, because up to 20% of patients are not seropositive.^24^ On the contrary, some patients show only APCA positivity without gastric histopathological changes, but subsequently develop gastric body atrophy. These patients are defined as “potential AIG.”^3^ The idea of “potential AIG” is well-established in gluten enteropathy, but is relatively new for AIG, where data on its natural progression, the rate of transformation into overt AIG, and its risk factors remain to be elucidated. To address this, Lenti et al^25^ followed 51 potential AIG patients for up to 15 years and found that approximately half of these patients progressed to AIG.

Of note, the older patients with AIG present with different symptoms as opposed to the younger patients. In order to clarify this issue, Kalkan and Soykan^26^ evaluated 119 patients with AIG older than 65 years and compared them with the AIG patients (n = 236) younger than 65 years of age. They reported some important divergences in the clinical and laboratory findings between those 2 groups. Older patients were more frequently evaluated for vitamin B_12_ and iron deficiencies. Polyautoimmunity and multiple autoimmune syndromes were more prevalent in older AIG patients, who also exhibited higher serum gastrin and chromogranin A levels.

### Histopathological and Laboratory Findings

Diagnosing AIG primarily relies on the histopathological examination of gastric biopsy specimens. At least, a total of 5 biopsy specimens should be obtained, including the antrum (2), the angular incisure (1), and the gastric body (2) in patients undergoing upper GI endoscopy. From the histological perspective, AIG is marked by chronic inflammation and progressive damage to the oxyntic glands, along with the parietal and zymogenic cells, primarily impacting the fundus and body mucosa.^27^ Histopathologically, AIG can be categorized into 3 stages: early phase, florid phase, and final stage.^28^ Int the beginning of the disease, lymphocyte and plasma cell infiltration is widespread or clustered within the deeper layers of the mucosa. During the florid stage, lymphocyte infiltration becomes more intense, leading to the disappearance of fundic glands and significant atrophy. As the condition progresses to its final stage, the changes observed in the florid phase continue, and the majority of the fundic glands vanish. Once the autoimmune activity’s target disappears, inflammation diminishes and inflammatory cell infiltration ceases. As fundic gland cells disappear, mucus cell compensation begins, resulting in pseudopyloric gland metaplasia. As fundic glands are lost, they are substituted by real pyloric metaplasia, intestinal metaplasia, and pancreatic acinar metaplasia. Additionally, due to the trophic effects of gastrin secreted in response to the parietal cell damage, ECL cell proliferation occurs, leading to a histological pattern known as ECL cell hyperplasia. This hyperplasia starts as a linear pattern, progressing to a tubular form, and eventually becoming nodular as inflammation advances. Nodular ECL cell hyperplasia, also known as endocrine cell micronests, is considered neoplastic when the nodular proliferation reaches a maximum size of 0.5 mm.^29^ This inflammatory process primarily takes place in the lamina propria. During the active phase, the crypt epithelium remains relatively intact, resulting in a high crypt epithelium-to-intrinsic gland ratio. This ratio is a key distinguishing feature in the histological picture of chronic gastritis caused by *H. pylori* infection, where inflammation starts within the glandular epithelium.

In a recent study by Miceli et al, AIG patients were classified into 5 stages based on histopathological findings: stage 0 represents potential AIG, in which the patients are APCA positive but without atrophy or metaplasia; stage 1 is the early stage, marked by lymphocytic and plasma cell infiltration with mild atrophy in the oxyntic mucosa; stage 2 is the florid stage, showing moderate atrophy with continued lymphocytic and plasma cell infiltration; stage 3 is the end stage, characterized by severe atrophy of the oxyntic mucosa; and stage 4 is the complicated stage, involving ECL cell dysplasia, type I NET, low- or high-grade nonendocrine dysplasia/glandular intraepithelial neoplasia, or adenocarcinoma.^30^

Although serum gastrin levels and the presence of APCA are complementary tests for the diagnosis of AIG, the occurrence of APCA was not deemed essential for verifying AIG, as up to 20% of patients are not seropositive.^24^ There are some non-invasive serological markers for the diagnosis of AIG that should be ordered before gastroscopy and biopsy. These are serum gastrin level, pepsinogens, and anti-Hp IgG, which is called a “serological biopsy.” Serum pepsinogen I & II can be used as markers for AIG. Pepsinogen I is secreted from the chief cells of the oxythic mucosa and pepsinogen II is secreted from both oxyntic and antral mucosa. In AIG, due to atrophy of the gastric body mucosa, pepsinogen I secretion is diminished. Low levels of pepsinogen I (<70 µ/mL), or a low ratio of pepsinogen I/II (<3), indicate the existence of body atrophy. Pooling data from the 7 studies that evaluated the performance of panel tests (serum gastrin, pepsinogen I & II, and anti-Hp IgG) for the diagnosis of body-related atrophic gastritis, the sensitivity and specificity were 70.4% and 98.4%, respectively.^31^

### Endoscopic Findings

**Figure 1.**
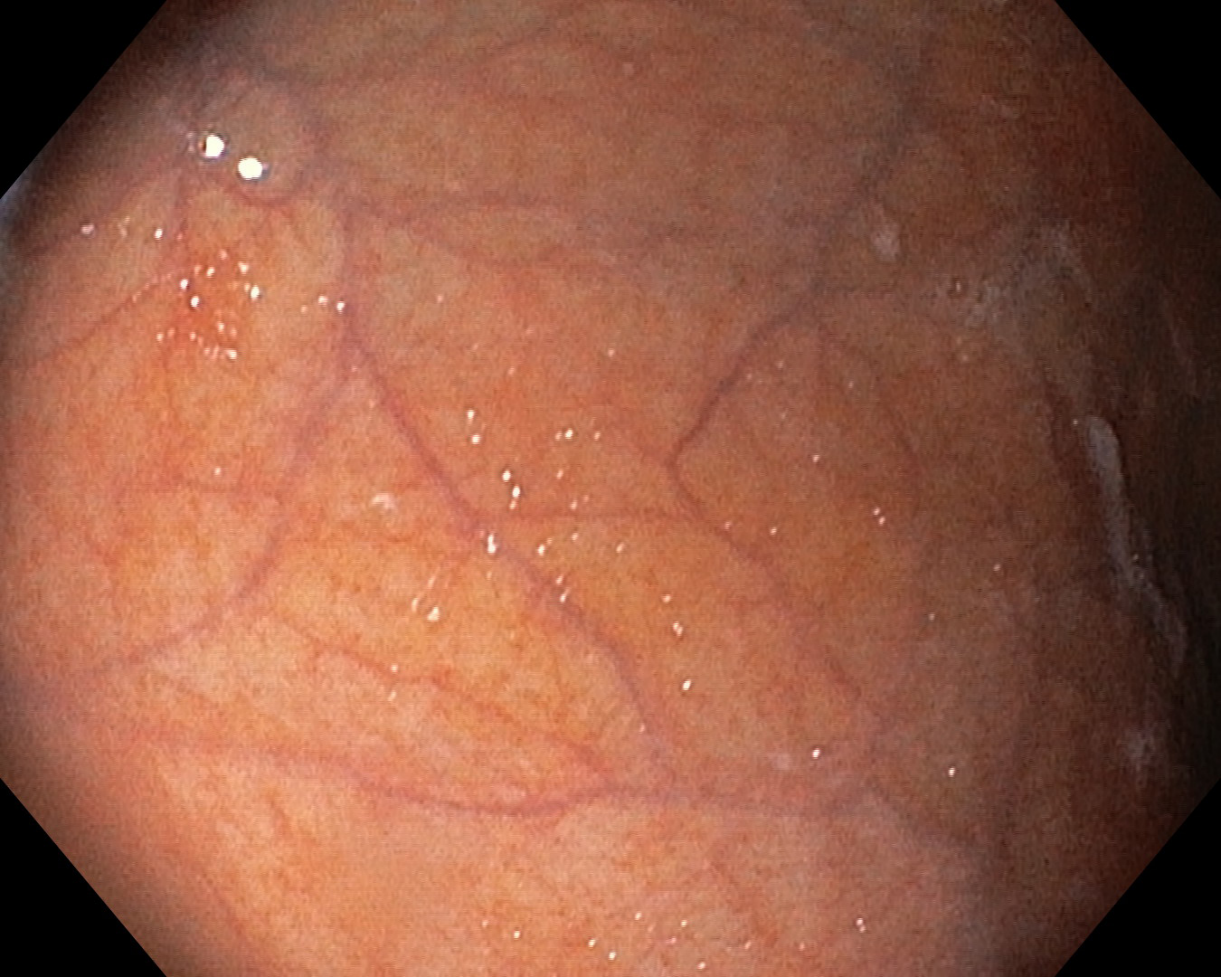
Endoscopic view of atrophy of the corpus mucosa showing the absence of rugal folds and submucosal vessels.

**Figure 2.**
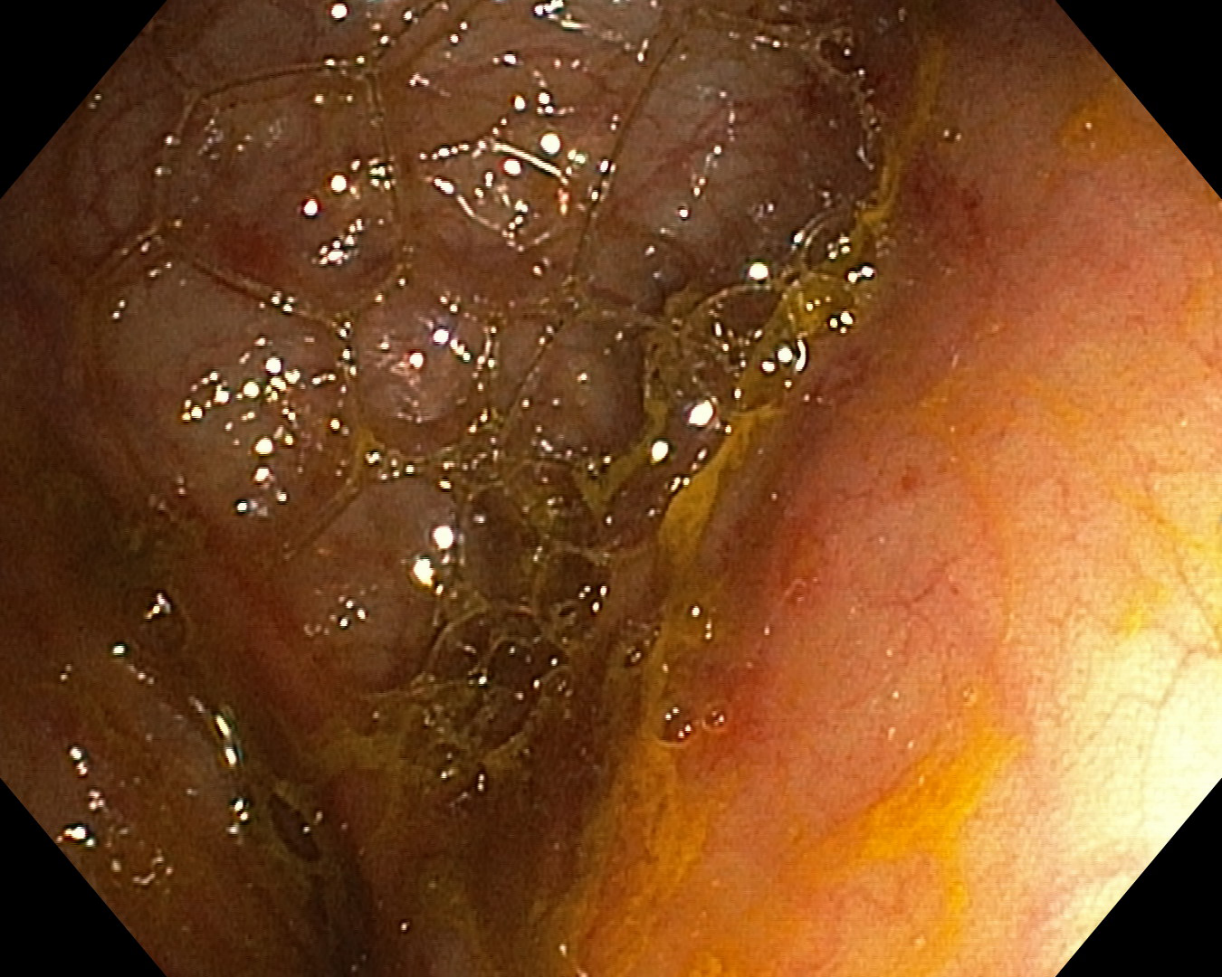
Sticky, dense, adherent mucus is the newest endoscopic finding described in patients with autoimmune gastritis. It is a yellow-to-white mucus that cannot be easily removed from the mucosa with water injection.

**Figure 3.**
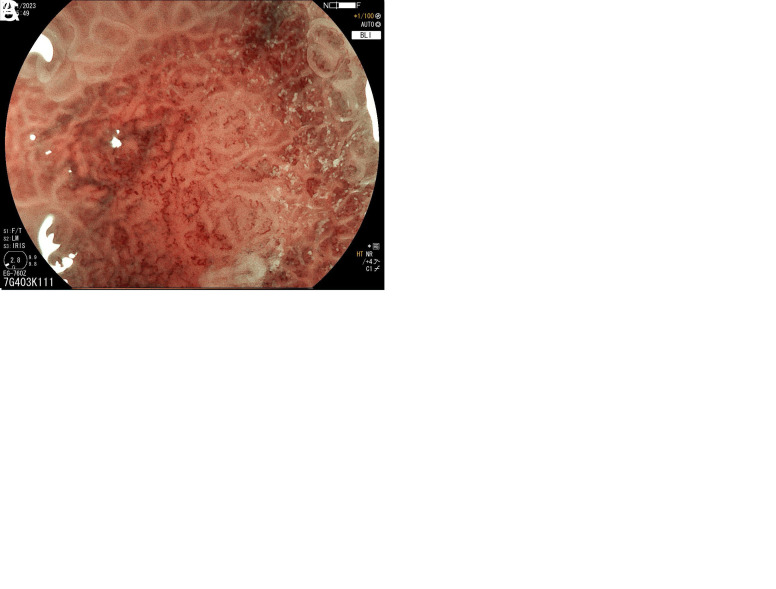
(A) A small neuroedocrine tumor type 1 with erosions on its top is seen in standart white light. (B) The same lesion is demonstrated by linked color imaging, which shows mucosal microstructure irregularities. (C) BLI of the same lesion showing abnormal findings of microstructure ande microvasculature in a close-up view.

The typical endoscopic finding in AIG is gastric body atrophy without antral involvement (Figure 1). Other endoscopic signs include remnant oxyntic mucosa, sticky adherent dense mucus, and hyperplastic polyps. These features are very important in the diagnosis of AIG, but they are seen in the middle to late stages rather than in the early stage.^32^ Terao et al^33^ analyzed the clinical and endoscopic features of 245 AIG patients in a multicenter study. In addition to the well-known body-dominant atrophy, containing submucosal vessel visibility and flattened rugal folds, one important finding of this study was the assessment of remnant oxyntic mucosa in the body. They categorized the remnant oxyntic mucosa into 5 types and assessed the prevalence of each. Another significant observation was the endoscopic picture of the antral mucosa. While it is generally expected that the antrum appears normal in AIG patients, this study found that fewer than 50% of the patients showed a normal pattern (i.e., mucosa without endoscopic atrophy). Autoimmune gastritis causes atrophy over a broad area of the oxyntic glands, although some normal fundic glandular mucosa may still be present. Krasinskas et al^34^ noted that multiple polyps containing gastric-acid-secreting tissue can develop in the atrophic mucosa of AIG cases, presenting as oxyntic mucosa pseudopolyps. This observation aligns with findings by Terao et al, indicating remnants of the oxyntic mucosa, particularly associated with pseudopolyp-type mucosa.^35^ A common endoscopic finding in AIG is hyperplastic polyps.^14^ In the later stages of AIG, multiple polyps may form in the gastric body. Terao et al^33^ found that gastric hyperplastic polyps were present in 21.2% of AIG cases.^33^ In a recent multicentric cross-sectional study by Massioroni et al, they enrolled 612 AIG patients who had undergone at least 1 endoscopic examination in order to define gastric polyp occurrence.^35^ They followed-up 612 patients for a median period of 4 years, and during this time, 222 of them (36.3%) developed at least 1 gastric polyp. Among these, 214 were non-endocrine lesions found in 162 patients, including 151 inflammatory polyps (70.5%), 29 adenomatous polyps (13.6%), 18 fundic gland polyps (8.4%), 13 adenocarcinomas (6.1%), and 1 MALT lymphoma. Additionally, gastric nNETs were identified in 108 patients, 48 of whom also had non-endocrine polyps. Factors such as older age and elevated levels of gastrin and chromogranin A were linked to polyp development. However, no important changes were noted in OLGA/OLGIM stages or *H. pylori* status between patients with and without lesions. In patients with AIG, the most common endoscopic appearance is sticky, adherent, and dense mucus (Figure 2). This type of sticky, adherent, and dense mucus adheres from the fundus to the upper part of the stomach, is pale yellow to white, and cannot be easily removed with water. This was observed in 32.4% of patients. The existence of sticky, dense mucus is associated with the presence of urease-producing bacteria other than *H. pylori* in achhorhydric state.^33^ Furthermore, these bacteria may cause false positives in *H. pylori* urea breath tests, potentially leading to failure in eradication therapy.^36^ Endoscopic changes were also found in the antrum of AIG patients. Terao et al^33^ reported “patchy redness” in 22.1% of cases, a “circular wrinkle-like pattern” in another 22.1%, “red streaks” in 10.4%, and “raised erosion” in 3.6%. These endoscopic findings in the antral mucosa suggest a variety of mucosal conditions, rather than just the presence of normal mucosa. Over the past 20 years, image-enhanced endoscopy has made significant advancements, with technologies such as narrow band imaging, flexible spectral imaging color enhancement, and i-Scan. Despite this progress, high-magnification imaging of irregular microvessels in malignant lesions remains insufficient. Narrow band imaging, in particular, has some drawbacks, including producing dark images of distant lesions in large luminal organs and offering poor visualization of the mucosal microstructure on tumor surfaces, making it less suitable for screening endoscopy. To address the limitations of current image-enhanced endoscopy methods, linked color imaging and blue laser (light) imaging systems have recently been developed. With the press of a single button during endoscopy, clinicians can easily switch between incorporating linked color imaging and blue laser (light) imaging systems. These systems improve both screening and detailed observation of the gastrointestinal tract by offering a longer observable distance than narrow band imagingI (Figure 3A and C).

There is scant data regarding the endoscopic signs in patients with early stage AIG. Kotera et al^37^ investigated 12 early-stage AIG cases by means of endoscopic images. They found “bamboo joint-like appearance” in 9 patients and a “salmon roe-like appearance” in 7 patients in the body of the stomach and non-atrophic or slightly atrophic antrum. They proposed that endoscopic appearances resembling bamboo joints or salmon roe could be indicative of early stage AIG. Additionally, the same research group described early stage AIG histological findings, such as intact parietal cell/mucous neck cell layer of the oxyntic glands, degeneration and pseudohypertrophy of the remaining parietal cells, lymphocytic infiltration between the oxyntic glands, and ECL cell hyperplasia that is not consistently observed.

### Consequences of Hypo/Achlorhydria and Therapeutic Considerations

The management of the patients with AIG focuses on correcting iron and vitamin deficiencies, as well as ensuring the early detection of pre-neoplastic lesions through regular endoscopic monitoring.

### Iron Deficiency

Hydrochloric acid secretion is mandatory for the conversion of non-heme iron from the ferric form to the ferrous form in order to be transported into the enterocytes. In cases of hypo/achlorhydria, iron deficiency occurs due to impaired dietary iron dissolution and inefficient absorption. Therefore, iron deficiency anemia may be the first clinical finding of AIG, being present in up to 50% of patients and serving as a clue for diagnosing AIG.^38^ Due to hypochlorhydria and impairment of iron absorption, oral iron therapy is not efficacious in AIG patients. For these reasons, therapy for iron deficiency should include the use of intravenous iron treatment.^39^ Therefore, it is very important to monitor iron status in these patients periodically.

### Vitamin B_12_ Deficiency

Parietal cells are responsible for HCl and intrinsic factor secretion. In AIG, atrophy of the oxyntic mucosa causes a reduction in the absorption of vitamin B_12_ due to hypochlorhydria and intrinsic factor deficiency. Moreover, the presence of HCl is very important in the splitting of vitamin B_12_ from food. In this condition, decreased absorption of vitamin B_12_ occurs, leading to vitamin B_12 _deficiency because of reduced secretion of HCl and intrinsic factor. In cases of vitamin B_12_ deficiency, it is suggested that vitamin B_12_ supplementation should be given via intramuscular or sublingual routes on a regular basis.^40^

### Gastric Flora

The stomach is not suitable for bacterial growth due to its low pH and acidic environment. In case of hypochlorhydria, the gastric mucosal barrier is altered and this may support bacterial overgrowth. Conti et al^41^ showed that in AIG patients, gastric flora showed higher colonization of “*Firmicutes*” particularly *Streptococcus*, which were also increased in individuals with severe atrophy/intestinal metaplasia, putting them at higher risk of gastric carcinoma. Thus, hypochlorhydria seen in AIG patients promotes the growth of these bacteria, for which a role in the pathway of carcinogenesis has been hypothesized.^42^

### Gastric Neuroendocrine Tumors and Gastric Adenocarcinoma

There are 3 main types of gastric NETs: type 1, type 2, and type 3. These tumors are also classified as either well-differentiated or poorly differentiated neoplasms. Well-differentiated tumors are further categorized as G1, G2, or G3 based off of their Ki-67 proliferation index or mitotic count as follows:^43-45^

G1: Proliferation index <3% or mitotic count <2/2 mm^2^

G2: Proliferation index: 3%-20% or mitotic count 2–20/2 mm^2^

G3: Proliferation index >20% or mitotic count >20/2 mm^2^

Type 1 tumors represent the majority of gastric NET cases, accounting for nearly 70%-80% of all cases.^46^ Abnormal proliferation of ECL cells causes gastric NETs, which are stimulated by gastrin.^47^ Consequently, type 1 NETs pose a significant dilemma for AIG, with an annual cumulative risk of about 5.7%.^48^ Gastric NETs are typically tiny polyp-like lesions, reported to be multiple in 65% of cases, mainly located in the gastric body or fundus, and confined to the mucosa or submucosa.^49^ Type 1 gastric NETs are generally well-differentiated, characterized by a Ki-67 index of less than 1%, and have a metastasis risk of less than 5%.^43^

Treatment options include endoscopic surveillance, endoscopic resection, surgical intervention, drug therapies (such as somatostatin analogs, targeted therapies, and chemotherapy), and peptide receptor radionuclide therapy. The choice of treatment largely depends on the patient’s clinical situation, the nature of the tumor as assessed by endoscopy, radiological and histologic findings, as well as the physician’s expertise. Exarchou et al reported that over 90% of type 1 gastric NETs smaller than 10 mm do not progress to more aggressive forms during prolonged endoscopic follow-up in a study conducted at 2 referral centers for neuroendocrine neoplasms.^50^ According to the 2023 European Neuroendocrine Tumor Society (ENETS) guidelines, type 1 gastric NETs under 10 mm and G1 can be actively followed-up without the need for frequent biopsies unless unusual features arise, such as ulceration, erosion, or pitting.^45^ Gastric NETs that are 10 mm or larger and/or classified above G1 should be regarded for resection. Larger lesions and those with higher Ki-67 levels have demonstrated a higher risk of local and distant metastasis, with the exception of low-grade G2 gastric NETs.^51^ Endoscopic ultrasonography should be employed before resection of tumors exceeding 10-15 mm or those not classified as G1 to evaluate local invasion depth and lymph node involvement, confirming their suitability for resection.^52^ However, European Neuroendocrine Tumor Society guidelines indicate that invasion of the muscularis propria is a contraindication for endoscopic resection.^45^ Additionally, ESGE guidelines advise endoscopic surveillance for AIG patients, with biopsies taken from the antrum and body every 3-5 years. In order to make a high-quality assessment, high-definition endoscopy combined with virtual chromoendoscopy should be employed.^53^ Patients with type 1 lesions who are under active surveillance should undergo upper endoscopy every 6 months during the first year following diagnosis, and then every 1-2 years if the disease remains stable. For those who have been resected for a type 1 NET, an annual endoscopic follow-up is recommended. However, a shorter interval may be advisable if there are risk factors such as incomplete resection, G2 grading, or a tumor size greater than 20 mm.^45^

There are several pharmacological therapeutic options for type 1 NETs for patients who are not suitable for surgery or endoscopic resection. One of them is a gastrin-receptor antagonist named netazepide. In a clinical trial, netazepide therapy resulted in a complete response in 30% of patients. However, after stopping the drug, all patients experienced tumor relapse.^54^ Type-1 NETs express somatostatin receptors due to the well differentiation of the tumor.^43^ In a recent systematic review, Rossi et al^55^ reported that management with somotostatin analogs for type-1 NETs showed an important response rate of approximately 25%-100%. Octreotide LAR (long-acting release) and lanreotide are the most commonly used drugs, at the usual dose of 30 mg/3-4 weeks and 60 mg/3-4 weeks, respectively, and it is better to evaluate the patient 12 months later to see if there is a response to the therapy.

There are several studies investigating the risk of gastric adenocarcinoma development in patients with AIG. Patients with AIG have a threefold increased risk of developing gastric adenocarcinoma compared to the general population.^56,57^ Rugge et al. evaluated the risk of gastric cancer in 211 AIG patients and followed them up for a mean of 7.5 years.^58^ During the follow-up period, no gastric adenocarcinoma was detected, and they concluded that AIG patients without incomplete intestinal metaplasia did not carry the risk of developing gastric adenocarcinoma. In a report by Miceli et al. investigating the long-term natural history of 498 AIG patients, epithelial dysplasia developed in 18 patients but no cases of gastric adenocarcinoma occurred, and they concluded that although AIG is a progressive disorder, the risk of developing gastric adenocarcinoma is negligible.^30^ Despite these findings, European guidelines accept AIG as a precancerous condition and suggest that AIG patients should be monitored endoscopically at intervals of 3-5 years.^59^

Autoimmune gastritis is a substantially benign situation when iron/vitamin B_12_ deficiencies are immediately corrected. Patients with autoimmune gastritis are often asymptomatic, and clinical suspicion is crucial in recognizing the signs and symptoms, such as iron and vitamin B_12_ deficiencies, to prevent diagnostic delays. Diagnosis of autoimmune gastritis still relies on the pathological examination of gastric body biopsies taken from the body mucosa, and it can be reinforced by non-invasive serological biomarkers such as gastrin, APCA, and pepsinogens. There are no specific treatment modalities for this disorder, and the therapy goal for patients with AIG is to restore iron and vitamin B_12_ deficiencies and to prevent neoplastic transformation through appropriate surveillance techniques.

## Data Availability

The data that support the findings of this study are available on request from the corresponding author.
